# Activation of NPY2R-expressing amygdala neurons inhibits itch behavior in mice without lateralization

**DOI:** 10.21203/rs.3.rs-4463812/v1

**Published:** 2024-05-24

**Authors:** Darya Pavlenko, Zeynep Todurga Seven, Kristen Sanders, Anika Markan, Rebecca Verpile, Hirotake Ishida, Dylan Costco, Tasuku Akiyama

**Affiliations:** 1 Dr. Phillip Frost Department of Dermatology & Cutaneous Surgery and Miami Itch Center, University of Miami Miller School of Medicine, Miami, FL, USA; 2 Department of Medical Pharmacology, Cerrahpasa Medical Faculty, Istanbul University-Cerrahpasa, Istanbul, Turkey

**Keywords:** Pruritus, Pain, Optogenetics, NPY, scratching, amygdala

## Abstract

The central amygdala (CeA) is a crucial hub in the processing of affective itch, containing a diverse array of neuronal populations. Among these components, Neuropeptide Y (NPY) and its receptors, such as NPY2R, affect various physiological and psychological processes. Despite this broad impact, the precise role of NPY2R^+^ CeA neurons in itch modulation remains unknown, particularly concerning any potential lateralization effects. To address this, we employed optogenetics to selectively stimulate NPY2R^+^ CeA neurons in mice, investigating their impact on itch modulation. Optogenetic activation of NPY2R^+^ CeA neurons reduced scratching behavior elicited by pruritogens without exhibiting any lateralization effects. Electrophysiological recordings confirmed increased neuronal activity upon stimulation. However, this modulation did not affect thermal sensitivity, mechanical sensitivity, or inflammatory pain. Additionally, no alterations in anxiety-like behaviors or locomotion were observed upon stimulation. Projection tracing revealed connections of NPY2R^+^ CeA neurons to brain regions implicated in itch processing. Overall, this comprehensive study highlights the role of NPY2R^+^ CeA neurons in itch regulation without any lateralization effects.

## Introduction

Itch, an uncomfortable sensation prompting scratching, is a common experience for most individuals. Beyond its physical symptoms, itch often causes or exacerbates stress and anxiety, leading to a significant reduction in quality of life for chronic itch patients^1^. Studies in both humans and mice have illuminated the involvement of the amygdala in both itch and anxiety^2–6^. Specifically, activating itch-responsive neurons in the central amygdala (CeA) has been shown to enhance both scratching behavior and anxiety-like responses in mice^2^.

The role of CeA in modulating itch is intricate, with evidence suggesting its involvement in both upregulating and downregulating this sensation^2,7,8^. This complexity likely stems from the diverse subpopulations of neurons within the CeA, predominantly composed of GABAergic neurons that project to numerous other brain regions^9^. Therefore, exploring the unique roles of CeA neuronal subsets becomes essential for understanding their impacts on itch, pain, and anxiety.

Neuropeptide Y (NPY) is widely distributed throughout the central and peripheral nervous systems. Vertebrates possess seven distinct NPY receptors, with four (NPY1R, NPY2R, NPY4R, and NPY5R) confirmed to be active in humans, all functioning as G-protein-coupled receptors^10,11^. NPY exerts a complex influence on the amygdala, impacting various processes such as anxiety, depression, stress response, alcohol consumption, food intake, and fear learning through receptor-mediated mechanisms^12–15^. Notably, the CeA predominantly expresses NPY2R and NPY1R^16,17^.

Further complexity arises from findings of lateralization in pain regulation within the CeA. Pro-nociceptive functions have been linked to the right CeA across various pain models, while the left CeA appears to have minimal impact on pain modulation^18^. However, research into the lateralization of CeA function in itch regulation remains limited.

In this study, we selectively stimulated NPY2R^+^ CeA neurons using the Npy2r-Cre mouse line, expressing the light-sensitive cation channel ChrimsonR in these neurons. Our aim was to explore the roles of NPY2R^+^ CeA neurons in itch regulation and investigate potential lateralization effects.

## Methods:

### Mice:

All mouse procedures were approved by Institutional Animal Care and Use Committees of the University of Miami (protocol number: 21–111), and were performed in accordance with the guidelines for the Care and Use of Laboratory Animals. The study was carried out in compliance with the ARRIVE guidelines.

Npy2r-Ires-Cre (*Npy2r*^*tm1.1(cre)Lbrl*^; strain#029285) and C57BL/6J mice were obtained from The Jackson Laboratory (Bar Harbor, ME). The mice were group housed (2–5 per cage) in a 12–12 light-dark cycle and had access to food and water *ad libitum*. Prior to all testing mice were habituated for 5 days to the testing room, handling, intradermal (i.d.) injections, and cable attachment. A small patch of fur on the nape of the neck was shaved for i.d. injections in all mice. Adult male and female mice were randomly assigned to experimental groups. No sex differences were noted for any quantified data. Therefore, sexes of the same genotype were pooled for analysis. All mice included in the study were over 8 weeks old at the time of surgery. Following surgery, mice were allowed a two-week recovery period prior to starting behavior testing. Mice were typically used for a battery of behavioral tests, with a one-week break between each test.

### Intra-CeA injection:

C57BL/6J mice were anesthetized with sodium pentobarbital (65 mg/kg, i.p.) and were positioned in the stereotaxic frame. Stereotaxic coordinates were determined according to the brain atlas by Paxinos and Watson: 1.34 mm posterior, and 4.5 mm lateral to bregma. Custom made guide cannulas (23G, Covidien, Dublin, Ireland) were inserted through small holes drilled into the skull. A separate hole was drilled into the contralateral position of the cannula insertion site on the skull. Dental cement was applied to the skull for fixation of the guide cannulas. The injector (33G, Practicon Inc, Greenville, NC) extended 4.5 mm beyond the skull. Two-week post-surgery, the injector connected to a polyethylene microtube was inserted through the guide cannula. The polyethylene microtube was connected to a Hamilton syringe. PYY3–36 (1 pmol or 10 pmol; Bio-Techne Corp., Minneapolis, MN) or its vehicle PBS was infused using the Hamilton syringe (250 nl/min) over 60 secs. Behavior was recorded for 30 minutes after injection and was analyzed by counting the number of scratches during 5-minute batches over 30 minutes by a blinded experimenter. After the behavioral tests, mice were sacrificed to verify cannula placements.

### Adeno-associated virus injection:

Npy2r-cre mice were anesthetized with sodium pentobarbital (65 mg/kg, i.p.) or isoflurane (3%) and placed into the stereotaxic frame. An adeno-associated virus (AAV) encoding a Cre-dependent fast opsin (ChrimsonR^19^) fused to enhanced red fluorescent protein (AAV5/Syn-FLEX-ChrimsonR-tdTomato; Addgene; a gift from Ed Boyden, MIT; Addgene viral prep #62723-AAV5; RRID:Addgene_62723) was unilaterally injected into the CeA [coordinates: anterior-posterior (AP) −1.34 mm, medial-lateral (ML) ±2.7 mm, dorsal-ventral (DV) −4.5 mm]. Control mice received unilateral injections of AAV5/Syn-FLEX-tdTomato [a gift from Hongkui Zeng (Addgene plasmid #51505; RRID:Addgene_51505)^20^. The injection volume of 0.25 μl was carefully administered over 60 secs using a glass needle and plunger. The viral dose was 8.5 × 10^12^ vector genomes/ml. For amygdala stimulation, an optic fiber (200 μm in diameter, 3.5 mm in length) was implanted directly above each injection site and fixed to the skull with dental cement. Mice were allowed two weeks to recover from surgery before behavior testing or electrophysiology.

### RNAscope:

C57BL/6J mice were anesthetized with sodium pentobarbital (80 mg/kg), transcardially perfused with 4% PFA, and dissected. Brains were flash-frozen in 2-methylbutane and stored at −80°C. Brain sections containing the CeA were sectioned coronally at 16 μm with a cryostat. Sections were prepared for hybridization per the manufacturer’s instructions (Advanced Cell Diagnostics, Inc, Newark, CA) using probes for *Npy2r* (Mm-Npy2r-C2, catalog # 315951-C2). Stitched photomicrographs of whole sections were obtained using fluorescence microscopy (Leica Microsystems, Wetzlar, Germany) at 10× objective magnification.

### Immunohistochemistry:

The brains of NPY2R-cre mice, following injection with AAV5/Syn-FLEX-ChrimsonR-tdTomato, were dissected and postfixed in 4% PFA for 4 h. Brains were cryoprotected in 30% sucrose for 24 h, frozen in Optimal Cutting Temperature compound (OCT; Sakura Finetek, Torrance, CA), and coronally sectioned at 20-μm thickness on a cryostat. Sections were stored in antifreeze solution at −20°C until staining.

For free-floating immunohistochemistry, sections were washed with PBS and then blocked with 5% normal goat serum in PBS with 0.2% Triton X-100 for 2 h at room temperature. Sections were incubated with mouse-anti-Cre primary antibody (1:300; MAB3120, Millipore Sigma, Burlington, MA; RRID:AB_2085748) in blocking buffer for 48 h at 4°C. Following primary incubation, sections were washed with PBS, incubated with goat-anti-mouse secondary antibody conjugated with Alexa Fluor 488 (1:300, Jackson ImmunoResearch Inc, West Grove, PA) in blocking buffer for 2 h at room temperature, washed again, and mounted on slides with VECTASHIELD Hard set Antifade Mounting Medium with DAPI (Vector Laboratories Inc, Newark, CA).

Stitched photomicrographs of whole sections were obtained using fluorescence microscopy (Leica Microsystems) at 20× objective magnification. Structural boundaries were delineated using ImageJ software, guided by established atlases^21^. The number of Cre+ neurons and/or TdTomato+ neurons in each region of interest was counted manually by a trained observer.

### Projection tracing:

Npy2r-cre mice injected with AAV5/Syn-FLEX-ChrimsonR-tdTomato were perfused with PBS followed by 4% paraformaldehyde. Mouse brains were dissected, postfixed in 4% paraformaldehyde overnight, and then transferred to 30% sucrose for 48 hours. Subsequently, the brains were frozen in OCT and cut at 40 μm. These sections were mounted on slides with VECTASHIELD Hard set Antifade Mounting Medium with DAPI (Vector Laboratories). Imaging was performed at 10x magnification using a fluorescent microscope (Leica). The acquired images were then meticulously analyzed to identify brain regions containing projections, with reference to a brain atlas for precise confirmation of the regions of interest.

### Optogenetic stimulation:

Npy2r-cre mice were anesthetized with isoflurane (3%), and flexible fiber patch cords were attached to the external ends of the optic fibers. Then, mice underwent a habituation period of 1 hour to acclimate to the cables. These patch cords were subsequently connected to an LED light source (Thorlabs Inc, Newton, NJ) that delivered yellow light (554 nm wavelength) capable of activating ChrimsonR. The light pulses were maintained at consistent intensity and frequency (2.0 mW, 2 Hz) via an LED driver connected to a waveform generator. Prior to implantation, the intensity of light was measured in each fiber using an optical power meter (Thorlabs).

### Scratch testing:

Mice were administered i.d. injections of 10 μL of histamine (50 μg, Sigma-Aldrich, St. Louis, MO), chloroquine (100 μg, Sigma-Aldrich), or serotonin (10 μg, Sigma-Aldrich) and then recorded for 30 minutes under conditions of either light ON or OFF. Each mouse underwent two trials for each pruritogen, with a month-long interval between repetitions of the same chemical. Prior to each testing session, the experimental chamber was sanitized with 70% ethanol, both at the beginning of the day and after completion of testing for each group of mice to minimize odor effects. The number of scratch bouts was analyzed in 5-min bins by a trained observer blinded to the treatment condition. One scratch bout was defined as one or more rapid back-and-forth hind paw motions directed toward and contacting the injection site, ending with licking or biting of the toes or placement of the hind paw on the floor. Hind paw movements directed away from the injection site (e.g., ear-scratching) and grooming movements were not counted^22^.

### Elevated Plus Maze:

The elevated plus maze (EPM) procedure followed established protocols^2,8^. Briefly, each mouse was placed into the center square of the EPM, facing an open arm, and video recorded for 9 min with recording divided into three 3-min epochs (OFF-ON-OFF or OFF-OFF-OFF). During the ON epoch, mice were stimulated with yellow light (2.0 mW, 2 Hz).

The time interval between experiments with OFF-ON-OFF and OFF-OFF-OFF conditions was four weeks. Videos were analyzed by a trained observer blinded to the treatment group. A mouse was considered to have entered an arm when all four paws were placed on the floor in that arm. Decreased time in open arms was a considered measure of anxiety-like behavior, and the total number of entries was used as a general measure of locomotion^23^.

### Von Frey test:

Mice were acclimated to a perforated metal floor for 120 min before testing. The plantar surface of the hind paws was tested with a series of von Frey filaments to determine the paw withdrawal threshold (PWT). This baseline PWT assessment was conducted prior to any light stimulation. Following a 30-minute interval, the mice were exposed to yellow light (2.0 mW, 2 Hz), and their hind paws were once again subjected to testing. The light stimulation was sustained throughout the testing session. The PWT was reassessed 60 minutes post-light stimulation. For the formalin test, after the baseline PWT assessment, mice received an i.d. injection of 20 μL of 2.5% formalin into the hind paw. Ninety minutes following the injection, PWT was reassessed under conditions of both light stimulation and no light stimulation. The strength (g) of the von Frey filament which induced paw withdrawal was noted for each stimulus.

### Hargreaves test:

Mice were habituated to the Hargreaves arena for 120 min before testing. To determine the paw withdrawal latencies (PWLs), the plantar surface of the hind paws was exposed to five heat trials at 5-min intervals. PWL was measured both at baseline and during yellow light stimulation (2.0 mW, 2 Hz). The light was turned on during each trial of the Hargreaves’ test. PWL was measured again 60 min after light stimulation. The beam active and idle intensities were 38 and 5, respectively. A cutoff of time of 10 s was used to prevent excessive tissue damage.

### Electrophysiology:

For *in vivo* single-unit recording from the amygdala, Npy2r-cre mice injected with AAV5/Syn-FLEX-ChrimsonR-tdTomato were anesthetized with urethane (1.5 g/kg, i.p.) and mounted in a stereotaxic frame. A craniotomy was performed above the amygdala, and a custom-made opto-electrode was inserted into the amygdala using the following stereotaxic coordinates: AP −1.34 mm; ML 2.7 mm; DV −4.5 mm. The distance between the optical fiber tip and the tungsten electrode tip was 1 mm. Unit activity was amplified and digitally displayed using Powerlab (A-D Instruments) and Spike2 software (CED Instruments). Action potentials were sorted by spike size and waveform and quantified as number of impulses per second. After a unit was isolated, the firing of the unit was recorded in response to the yellow light stimulation. At the conclusion of the experiment, an electrolytic lesion was made at the recording site, and brains were processed for sectioning.

### Statistical analysis:

Results are presented as mean ± SEM. For comparison between two groups, a two-tailed Student’s t test was used. For comparison among more than two groups, a one-way RM ANOVA followed by Tukey’s multiple comparisons test or a one-way ANOVA followed by Dunnett’s multiple comparisons test was used. Statistical significance was set at *p* < 0.05 for all tests. All statistical analyses and graphs were made using GraphPad Prism9 (GraphPad Software, San Diego, CA).

## Results:

### Optogenetic stimulation increases the firing rate of NPY2R^+^ neurons.

**Figure 1.**
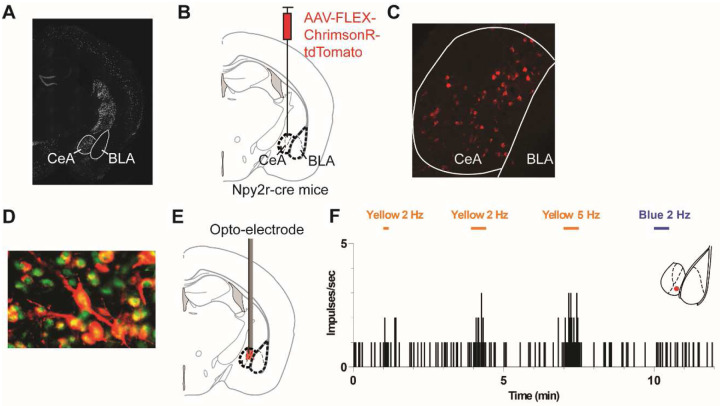
Optogenetic stimulation excited NPY2R^+^ neurons in the central nucleus of the amygdala (CeA). (A) A representative image of coronal section of amygdala displays *Npy2r* mRNA signals detected by RNAscope in the CeA of C57BL/6 mice. (B-C) Npy2r-cre mice received unilateral injections of AAV viruses containing the Cre-dependent viral construct (AAV-FLEX-ChrimsonR-tdTomato) or control (AAV-FLEX-tdTomato) into the CeA. Schematic drawing shows a mouse brain coronal section (Bregma: −1.34 (Adapted from Franklin and Paxinos 2008), B). Representative image shows tdTomato expression in the CeA (C). (D) Representative image of coronal section of amygdala showing immunoreactivity for Cre (green) and NPY2R^+^ neurons (red) (E) Schematic of experimental insertion of opto-electrode in the CeA. (F) Responses of a single unit recorded from the CeA of Npy2r-cre mice expressing AAV-FLEX-ChrimsonR-tdTomato in response to different light stimuli. The unit displayed increased firing rates in response to yellow light stimuli. From left to right, the firing rate increased during 10-second duration of 2 Hz yellow light, 30-second duration of 2 Hz yellow light, and 30-second duration of 5 Hz yellow light. No significant response was observed during a 30-second duration of 2 Hz blue light stimulation. Recording site shown in inset.

Using RNAscope, we initially explored the spatial distribution of *Npy2r* mRNA within the amygdala. As depicted in Figure 1A, *Npy2r*^+^ cells exhibited a predominant localization within the CeA, which is consistent with previous reports^16,24^. Subsequently, to specifically express chrimsonR tagged with tdTomato in NPY2R^+^ CeA neurons, AAV-FLEX-chrimsonR-tdTomato was injected into the CeA of Npy2r-cre mice (Fig. 1B). As demonstrated in Figure 1C, cells expressing chrimsonR-tdTomato were primarily concentrated in the CeA region. To validate the Cre-dependent expression of chrimsonR-tdTomato, dual fluorescence imaging for Cre and tdTomato was conducted. TdTomato expression was detected in 70.5 % (79/112, *n*=5 sections from 2 mice) of Cre-expressing neurons, while almost all tdTomato-expressing neurons coexpressed Cre (96.3%, 79/82, *n*=5 sections from 2 mice; Fig. 1D).

Furthermore, to ascertain the functionality of ChrimsonR expressed in the NPY2R^+^ CeA neurons, *in vivo* single unit electrophysiological recordings were conducted (Fig. 1E). As anticipated, amygdala neurons expressing ChrimsonR exhibited heightened activity under yellow light stimulation, while no significant response was observed under blue light (Fig. 1F).

### Optogenetic stimulation of NPY2R^+^ neurons in the CeA reduces scratching behavior without lateralization.

**Figure 2.**
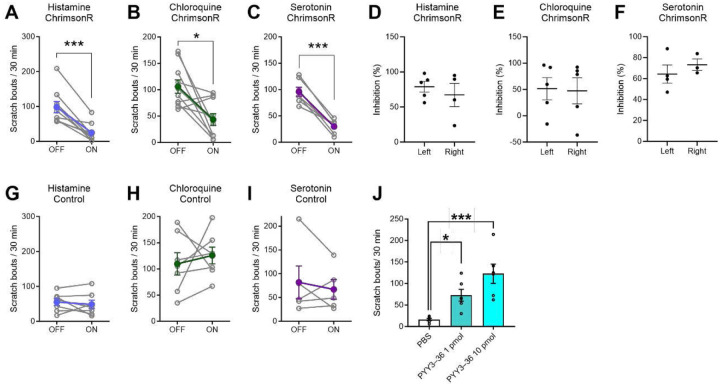
Optogenetic stimulation of NPY2R^+^ amygdala neurons inhibited itch-related behavior. (A) Number of scratch bouts per 30 min after i.d. injection of histamine, with or without concurrent light stimulation, in Npy2r-cre mice that received AAV-FLEX-ChrimsonR-tdTomato. Mice received ipsilateral intra-CeA injections of AAV-FLEX-ChrimsonR-tdTomato. Optic fibers were implanted above the CeA (n=9/group). (B) As in A, for i.d. chloroquine in Npy2r-cre mice that received AAV-FLEX-ChrimsonR-tdTomato (n=10/group). (C) As in A, for i.d. serotonin in Npy2r-cre mice that received AAV-FLEX-ChrimsonR-tdTomato (n=7/group). (D) Comparison was made between left and right stimulation, with data presented as percentages of inhibition relative to histamine-evoked scratching in the absence of light. (E) As in D, for chloroquine-evoked scratching. (F) As in D, for serotonin-evoked scratching. (G) As in A, for i.d. histamine in Npy2r-cre mice that received AAV-FLEX-tdTomato (control; n=6/group). (H) As in A, for i.d. chloroquine in control mice (n=6/group). (I) As in A, for i.d. serotonin in control mice (n=6/group). (J) Number of scratch bouts per 30 min after intra-CeA injection of PYY3–36 (1 or 10 pmol) or PBS (vehicle; n=8/group). Data are shown as mean ± SEM. *p < 0.05, ***p < 0.001, for paired *t* test versus light off (A-C). *p < 0.05, ***p < 0.001, for one-way ANOVA followed by Dunnett’s multiple comparisons test versus PBS (J).

To investigate the impact of optogenetic stimulation on NPY2R^+^ amygdala neurons on itch-related behavior, we recorded mouse responses following i.d. injections of the pruritogens histamine, chloroquine, and serotonin, with and without concurrent light stimulation. Light activation of NPY2R^+^ amygdala neurons notably decreased total scratching responses to each pruritogen (Fig. 2A,B,C; histamine, t(8)=5.674, *p*=0.0005; chloroquine, t(9)=3.154, *p*=0.0117; serotonin, t(6)=8.242, *p*=0.0002). Control mice subjected to identical light stimulation did not exhibit a significant reduction in scratch bouts triggered by the pruritogens (Fig. 2G,H,I).

Furthermore, to assess whether inhibition of scratching displayed laterality, we compared left and right-sided stimulation of NPY2R^+^ amygdala neurons. The reduction in scratching did not significantly differ between left and right stimulation (Fig. 2D,E,F; histamine, t(4)=0.6610, *p*=0.5558; chloroquine, t(5)=0.0973, *p*=0.9272; serotonin, t(3)=0.2461, *p*=0.8285).

In a complementary investigation into the role of NPY2R^+^ neurons in the CeA, we employed a pharmacological approach. Given that NPY2R receptors are linked to the Gi/o subunit and their activation leads to neuronal inhibition^10^, we hypothesized that intra-CeA administration of an NPY2R agonist would induce scratching by suppressing NPY2R^+^ neurons. Indeed, intra-CeA administration of PYY 3–36 resulted in a dose-dependent increase in scratching behavior without any external pruritogenic stimulation (Fig. 2J; F(2,15)=12.3, *p*=0.0007).

### Optogenetic stimulation of NPY2R^+^ neurons in the CeA did not change thermal sensitivity, mechanical sensitivity, or inflammatory pain.

**Figure 3.**
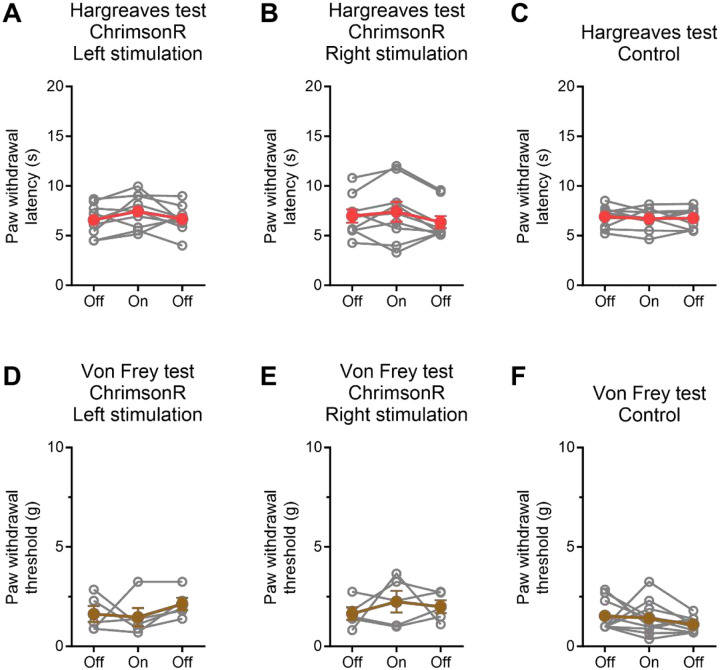
Optogenetic stimulation of NPY2R^+^ amygdala neurons did not affect basal thermal or mechanical sensitivities. (A) Paw withdrawal latency in Npy2r-cre mice that received AAV-FLEX-ChrimsonR-tdTomato. The Hargreaves test was performed before, during, and after left light stimulation (n=9/group). (B) As in A for right light stimulation (n=9/group). (C) As in A for Npy2r-cre mice that received AAV-FLEX-tdTomato (control; n=10/group). (D) Paw withdrawal threshold in Npy2r-cre mice that received AAV-FLEX-ChrimsonR-tdTomato. The von Frey filament test was performed before, during, and after left light stimulation (n=5/group). (E) As in A for right light stimulation (n=5/group). (F) As in A for Npy2r-cre mice that received AAV-FLEX-tdTomato (control; n=10/group). Data were analyzed using one-way repeated measure ANOVA followed by Tukey’s multiple comparison test. Error bars represent SEM.

Considering the central role of the CeA in pain processing^25,26^, we investigated whether optogenetic stimulation of NPY2R^+^ amygdala neurons affects thermal sensitivity, mechanical sensitivity, or inflammatory pain. The Hargreaves test and von Frey filament test were utilized to examine the impact of optogenetic stimulation of NPY2R^+^ amygdala neurons on basal thermal and mechanical sensitivities, as well as lateralization effects. In the Hargreaves test, neither left nor right-sided light stimulation of NPY2R^+^ amygdala neurons significantly affected withdrawal latencies (Fig. 3A–B; left, F(1.904,15.23)=1.672, *p*=0.2209; right, F(1.459,11.68)=2.325, *p*=0.1491). Similarly, in the von Frey filament test, neither left nor right-sided light stimulation showed a significant effect on paw withdrawal thresholds (Fig. 3D–E; left, F(1.204,4.815)=0.6996, *p*=0.4697; right, F(1.082,4.328)=0.6403, *p*=0.4777). Moreover, the same light stimulation exhibited no significant effect on either paw withdrawal latencies or thresholds in control mice (Fig. 3C, 3F).

**Figure 4.**
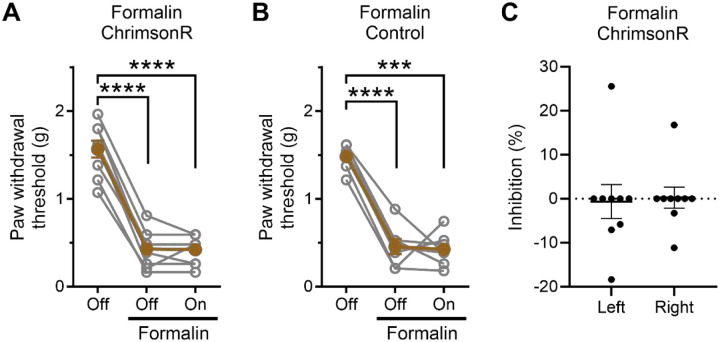
Optogenetic stimulation of NPY2R^+^ amygdala neurons did not affect formalin-induced mechanical hyperalgesia. (A) Formalin was injected into hind paw of Npy2r-cre mice that received AAV-FLEX-ChrimsonR-tdTomato. The von Frey filament test was performed before and after formalin with and without ipsilateral light stimulation (n=9/group). (B) Npy2r-cre mice that received AAV-FLEX-tdTomato (control; n=7/group). (C) Comparison was made between left and right stimulation, with data presented as percentages of inhibition relative to paw withdrawal threshold after formalin in the absence of light. Data are shown as mean ± SEM. ***p < 0.001, ****p < 0.0001, for one-way repeated measure ANOVA followed by Tukey’s multiple comparison test versus light off.

To evaluate the involvement of NPY2R^+^ amygdala neurons in inflammatory pain, we explored the effect of optogenetic stimulation on formalin-induced secondary mechanical hyperalgesia^27,28^. Despite formalin-induced decreases in paw withdrawal thresholds, light stimulation failed to restore these thresholds in both ChrimsonR-injected and control mice (Fig. 4A–B; ChrimsonR, F(1.154, 9.228)=180.6, *p*<0.0001; control, F(1.302, 7.812)=74.21, *p*<0.0001). Additionally, no lateralization effect was observed (Fig. 4C; t(8)=0.302, *p*=0.7694).

### Optogenetic stimulation of NPY2R^+^ neurons in the CeA failed to elicit alterations in anxiety-like behaviors or locomotion activity.

**Figure 5.**
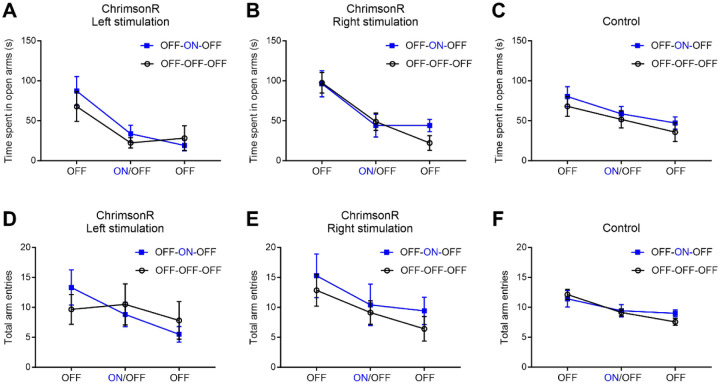
Optogenetic stimulation of NPY2R^+^ amygdala neurons did not affect anxiety-like behavior. Npy2r-cre mice received bilateral intra-CeA injections of either AAV-FLEX-ChrimsonR-tdTomato or AAV-FLEX-tdTomato (control). Optic fibers were implanted above the CeA. The EPM test was performed under light off and on conditions. (A) Left light stimulation did not affect open arm time in Npy2r-cre mice that received AAV-FLEX-ChrimsonR-tdTomato (n = 6/group). (B) As in A for right light stimulation (n = 7/group). (C) As in A for control mice (n = 7/group). (D) Left light stimulation did not affect the total number of arm entries, a measure of locomotion in Npy2r-cre mice that received AAV-FLEX-ChrimsonR-tdTomato (n = 6/group). (E) As in D for right light stimulation (n = 7/group). (F) As in D for control mice (n = 7/group). Data were analyzed using two-way repeated measure ANOVA followed by Bonferroni’s multiple comparison test. Error bars represent SEM.

Given the pivotal role of the CeA in processing itch- and pain-induced negative affect^2,29^, we investigated whether optogenetic stimulation of NPY2R^+^ amygdala neurons affects anxiety-like behavior and explored potential lateralization effects. To assess anxiety-like behavior, we employed the EPM, a standard tool for such evaluations. Neither left nor right-sided light stimulation resulted in a significant alteration in the time spent in the open arms (Fig. 5A–B; left, F(1,10)=0.2350, *p*=0.6383; right, F(1,12)=0.141, *p*=0.7138). This suggests that NPY2R^+^ amygdala neurons may not substantially contribute to anxiety-like behavior. Additionally, neither left nor right-sided light stimulation affected the total number of arm entries, indicative of locomotor activity (Fig. 5D–E; left, F(1,10)=0.001030, *p*=0.9750, right, F(1,12)=0.4016, *p*=0.5382). This result indicates that NPY2R^+^ neurons in the CeA do not play a major role in motor activity, which aligns with a previous finding demonstrating that genetic depletion of NPY2R in the CeA did not affect motor activity^16^. The same light stimulation showed no significant effect on either the time spent in open arms or total arm entries in control mice (Fig. 5C, 5F).

### NPY2R^+^ CeA neurons projected to the bed nucleus of the stria terminalis (BNST), ventrolateral periaqueductal gray (vlPAG), substantia nigra (SN) and parabrachial nucleus (PBN).

**Figure 6.**
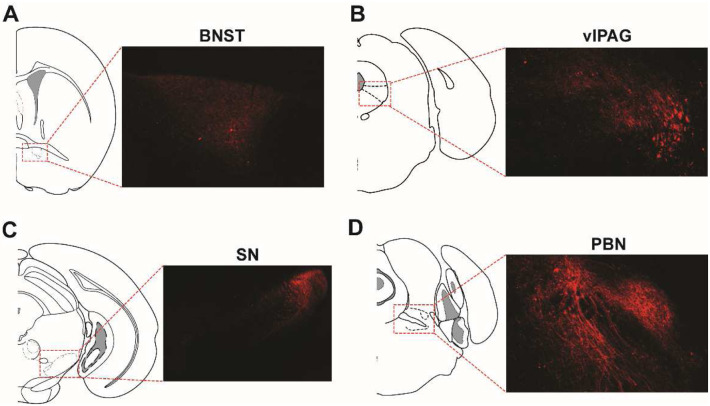
NPY2R^+^ amygdala neurons projected to brain regions involved in itch processing. Npy2r-cre mice received unilateral injections of AAV viruses containing the Cre-dependent viral construct (AAV-FLEX-ChrimsonR-tdTomato) into the CeA. Representative images depict tdTomato expression in the bed nucleus of the stria terminalis (BNST; A), ventrolateral periaqueductal gray (vlPAG; B), substantia nigra (SN; C) and parabrachial nucleus (PBN; D).

CeA neurons are known to project to key brain regions implicated in itch processing, such as the BNST, vlPAG, SN, PBN, and lateral hypothalamus (LH)^9,30^. To investigate whether NPY2R^+^ CeA neurons contribute to these pathways, brain sections from NPY2R-cre mice injected with AAV5/Syn-FLEX-ChrimsonR-tdTomato were examined. The analysis confirmed robust tdTomato expression in the BNST, vlPAG, SN, and PBN (Fig. 6). TdTomato expression was not observed in the LH.

## Discussion

In this study, we present compelling evidence supporting the role of NPY2R^+^ CeA neurons in reducing itch-related behavior. Notably, our investigation did not reveal any lateralization effect on itch inhibition by optogenetic stimulation of these neurons. Additionally, we showed that intra-CeA administration of an NPY2R agonist induces itch-related behavior by inhibiting NPY2R^+^ CeA neurons via Gi/o signaling. Furthermore, through projection tracing, we identified projections of NPY2R^+^ CeA neurons to brain regions known to be involved in itch processing. Collectively, these findings provide substantial support for the role of NPY2R^+^ CeA neurons in regulating itch signals.

We found that NPY2R^+^ CeA neurons project to the BNST, vlPAG, SN, and PBN. Interestingly, our previous research revealed that PDYN^+^ CeA-PBN-projecting neurons contribute to the regulation of itch-related behaviors. Notably, 32% of *Npy2r*^+^ CeA neurons express *Pdyn* (data analyzed from a single-cell RNA sequencing dataset, GSE213828)^9^. This suggests that CeA neurons co-expressing NPY2R and PDYN may play an important role in itch processing. Nonetheless, further research is needed to determine which projections are required for the CeA to process itch signaling.

Although lateralization of pain in the amygdala has been described, there is currently no evidence that itch processing is lateralized in this region. In a study of human volunteers, histamine was delivered via inactivated cowhage spicules to the left volar aspect of forearm and activated both the left and right amygdala^4^. In another investigation, histamine and cowhage were administered to the volar portion of the forearm unilaterally, again activating both the left and right amygdala^5^. Our results further support the notion that there is no evidence of lateralization of itch processing in the CeA.

The CeA is known to bi-directionally modulate pain through distinct subpopulations of neurons^26^. While PKCδ^+^ CeA neurons amplify pain, somatostatin^+^ (SST^+^) CeA neurons suppress it. *Prkcd* and *Sst* are expressed by 24% and 38% of *Npy2r*^+^ CeA neurons, respectively (data analyzed from a single-cell RNA sequencing data set, GSE213828)^9^. In this study, optogenetic stimulation of NPY2R^+^ neurons did not affect thermal sensitivity, mechanical sensitivity, or inflammatory pain. Co-activation of PKCδ^+^ and SST^+^ CeA neurons may counteract each other’s effects. Alternatively, the activation of small subsets of these neurons may be insufficient to affect pain perception.

The role of NPY2R^+^ amygdala neurons in anxiety-like behaviors appears to paint a complex picture. In previous reports, while the intra-CeA injection of an NPY2R antagonist reduced anxiety-like behavior, the intra-basolateral amygdala (-BLA) injection of an NPY2R agonist produced a similar effect^31,32^. However, the genetic depletion of NPY2R in the CeA or BLA, also led to a decrease in anxiety-like behavior^16^. Interestingly, in the present study, optogenetic stimulation of NPY2R^+^ CeA neurons had no significant effect on anxiety-like behavior. This suggests that direct activation of these neurons may not be the only factor determining anxiety-related behaviors. It is conceivable that optogenetic stimulation of NPY2R^+^ CeA neurons and disinhibition of these neurons via NPY2R may activate distinct neuronal populations, resulting in different behavioral outcomes.

In summary, our study highlights the potential of optogenetic stimulation of NPY2R^+^ CeA neurons in reducing itch signals. Additionally, we have mapped their connections to key brain regions involved in itch processing. Future investigations should focus on elucidating the specific contributions of these projections to itch signal processing.

## Data Availability

The datasets generated and analyzed during the current study are available from the corresponding authors upon reasonable request.
